# Training Allied Healthcare Professionals Maybe the Answer to Upscaling Gut‐Directed Hypnotherapy

**DOI:** 10.1002/ueg2.70051

**Published:** 2025-05-20

**Authors:** Dipesh H. Vasant, Jane Boissiere, Peter J. Whorwell

**Affiliations:** ^1^ Neurogastroenterology Unit Wythenshawe Hospital Manchester University NHS Foundation Trust Manchester UK; ^2^ Division of Diabetes Endocrinology and Gastroenterology University of Manchester Manchester UK; ^3^ British Society of Clinical and Academic Hypnosis Bridlington UK

Developed in Manchester over 40‐years ago [1], gut‐directed hypnotherapy (GDH) has become an established, highly effective, brain‐gut behavioural therapy (BGBT). GDH is one of the few treatments with proven benefit in refractory irritable bowel syndrome (IBS) [2]. Despite a rapid increase in interest, a considerable evidence‐base [3], and endorsement in IBS clinical guidelines around the world, access to GDH still remains limited to a relatively small number of specialised centres.

Recent efforts to improve access have aimed to address this with evidence supporting efficacy of shorter course durations of GDH [4], remote delivery [5] and group therapy [3]. Smartphone applications for GDH skills training may also have a role, especially in those with milder symptoms [6]. However, while these innovations have all shown promising results in optimising existing services, the lack of suitably trained therapists remains a concern, and a stumbling block to developing new GDH services. Meanwhile, multidisciplinary, integrated care for patients with IBS with access to medical, dietary and behavioural therapies such as GDH has quickly become the gold standard of care, with data suggesting that this approach not only improves clinical outcomes but also is cost‐effective [7]. One of the advantages of GDH is that it is a BGBT which can be delivered effectively by providers who are not necessarily medically or psychologically qualified but have been appropriately trained using validated protocols [2].

In this issue of *United European Gastroenterology Journal,* Lovdahl et al., have for the first time demonstrated the long‐term efficacy of nurse‐administered GDH [8]. In a 24‐month prospective follow‐up study of 207 patients with IBS using validated outcomes measures for gastrointestinal, somatic and psychological symptoms, the authors have demonstrated that 64.3% of those completing a 12‐week GDH programme (either via individual or group intervention) achieved clinical response. Follow‐up data at 6, 12 and 24 months, demonstrated remarkable stability in response rates [8]. Previous research has shown that GDH relieves extraintestinal and psychological symptoms [2, 4], with greater effects in those with more severe symptoms [9]. Interestingly, all these characteristics were observed in Lovdahl's study. In addition, Lovdahl et al. found that their response rates were greater in those that had individualised GDH and in younger patients. They speculated that response in younger patients may a reflection of shorter duration of disease. This would also help to explain why previous studies have demonstrated even greater response rates to GDH in children and adolescents with IBS [10].

The data from the Lovdahl et al. study suggests that training more nurses or allied healthcare professionals (AHPs) to administer GDH may be an attractive, cost‐effective solution to setting up GDH services alongside IBS clinics around Europe. Many nurses with a background in gastroenterology, or other AHPs including dieticians and GI physiologists, would be very well placed to learn core hypnotherapy skills and deliver GDH services. However, it is essential that those trained to administer GDH are under the strict supervision of a gastroenterologist who can act as a ‘gatekeeper’ selecting appropriate candidates for GDH with a secure IBS diagnosis. Furthermore, they can ensure broader psychological input for those with more complex mental health comorbidities and appropriate referral to psychiatric services, if necessary. This has been the model used in our Manchester unit where, over the years, where we have almost exclusively used therapists who have been nurses, AHPs or individuals with a degree in a biological science.

Training pathways for hypnotherapy vary around the world. In the United Kingdom where GDH originated, despite approval in the NICE guidelines for IBS, there are still only a handful of services with trained therapists equipped to provide GDH services in the National Health Service. The British Society of Clinical and Academic Hypnosis (BSCAH) is a small charity which is a Constituent Society of both the European Society of Hypnosis (ESH) and The International Society of Hypnosis (ISH). It has been running regular Medical Hypnosis courses for healthcare colleagues for many years with an option for a university accredited diploma. Recognising the need to train more AHPs to administer GDH, the BSCAH is now working with gastroenterologists and established UK GDH centres to develop a bespoke GDH course for AHPs consisting of both remote and face‐to‐face components, alongside continuing mentorship and a framework for clinical practice. Similar models in other countries may be the key to meeting the demand and upscaling access to this highly effective treatment.

## Data Availability

The authors have nothing to report.
